# Creep–Fatigue Experiment and Life Prediction Study of Piston 2A80 Aluminum Alloy

**DOI:** 10.3390/ma14061403

**Published:** 2021-03-13

**Authors:** Yi Dong, Jianmin Liu, Yanbin Liu, Huaying Li, Xiaoming Zhang, Xuesong Hu

**Affiliations:** 1 Vehicle Engineering Department, Army Academy of Armored Forces, Beijing 100072, China; 13391567396@163.com (Y.D.); ljm@163.com (J.L.); ss_sw_love@126.com (H.L.); true@163.com (X.Z.); 2 Department of Weapon and Control, Army Academy of Armored Forces, Beijing 100072, China; hxsaaaf@163.com

**Keywords:** piston, thermal and mechanical stress field, creep–fatigue experiment, support vector machine, cycle hysteresis energy

## Abstract

In order to improve the reliability and service life of vehicle and diesel engine, the fatigue life prediction of the piston in a heavy diesel engine was studied by finite element analysis of piston, experiment data of aluminum alloy, fatigue life model based on energy dissipation criteria, and machine learning algorithm. First, the finite element method was used to calculate and analyze the temperature field, thermal stress field, and thermal–mechanical coupling stress field of the piston, and determine the area of heavy thermal and mechanical load that will affect the fatigue life of the piston. Second, based on the results of finite element calculation, the creep–fatigue experiment of 2A80 aluminum alloy was carried out, and the cyclic response characteristics of the material under different loading conditions were obtained. Third, the fatigue life prediction models based on energy dissipation criterion and twin support vector regression are proposed. Then, the accuracy of the two models was verified using experiment data. The results show that the model based on the twin support vector regression is more accurate for predicting the material properties of aluminum alloy. Based on the established life prediction model, the fatigue life of pistons under actual service conditions is predicted. The calculation results show that the minimum fatigue life of the piston under plain condition is 2113.60 h, and the fatigue life under 5000 m altitude condition is 1425.70 h.

## 1. Introduction

In the working process, the piston of the diesel engine will be continuously subjected to the thermal stress caused by the thermal gradient, the mechanical stress applied by the piston pin, and the gas burst pressure in the cylinder under high-temperature conditions. The working environment of the piston is very bad, and fatigue failure is easy to occur. In addition, the working area of the vehicle is relatively complex, and the working conditions such as instantaneous acceleration, rapid braking, continuous output of high speed, and large load often appear in the working process, which leads to further deterioration of the working environment of the piston. At this time, the thermal–mechanical coupling stress close to the material limit will be generated in the local area of the piston. Working in this environment for a long time, the piston is prone to fatigue and creep failure, and other damages; therefore, it is necessary to study its working state and fatigue life in detail.

There are many studies that focus on the thermal and mechanical load of pistons. Rayapati Subbarao et al. [1] analyze the thermal and mechanical state of pistons using the finite element method by changing the working condition and alloy material of pistons. Results show that Alcoa Delt alloy is the best selection alloy for pistons. Potturi S. Prakash Varma et al. [2] use the computational fluid dynamics method to analyze the influence of different bowl geometry of piston on its combustion and soot character. At the end of the article, the authors come up with the developing trend about engine combustion and exhaust pollution. Fujun Huang et al. [3] analyze the working status and heat transfer condition of a free-piston engine by experimental and theoretical study. Results show that the main energy loss occurs at the process of exhaust. Akhil Reddy Anugu et al. [4] design a piston by considering its geometry and thermal loads. The authors compare two materials when designing the piston, and judge the performance of the piston by indicators of stress state, thermal field, and model accuracy. Qin Zhaoju et al. [5] analyze and optimize a piston in a diesel engine with finite element and multidisciplinary optimization methods. There are also some studies that focus on parameter study of piston [6], coated properties [7], diesel engine combustion [8], the geometry of piston [9], and stress analysis [10].

As for creep–fatigue experiment, the studies are focused on the loading rate sensitivity, viscoelastic, etc. In order to investigate the property and fatigue life of 316L steel, Wufan Chen et al. [11] conduct tensile, low cycle fatigue, and creep–fatigue experiments. Results from the monotonic tensile experiment show that the material is loading rate sensitivity. Mohammad Amjadi et al. [12] study and analyze the high-density polyethylene materials by experimental and theoretical models under different loading temperatures, loading frequencies, different stress, and different manufacturing techniques. Ultimately, they build the fatigue life prediction model based on the experiment data. Zeinab Y. Alsmadi et al. [13] study the creep–fatigue properties of 709 alloy by a series of strain-controlled experiments. The experiment conditions include that temperature is 650 °C and 750 °C, the tensile holding time is 0, 1, 10, 30, and 60 min, and strain range is 1% of the specimen. Results of the experiment show that the material exhibits dynamic strain aging property at 650 °C. Ultimately, they establish the creep–fatigue life prediction model based on the linear damage summation method. Yang Zhong et al. [14] study the creep–fatigue properties of 617alloy that works in the hard environment of 950 °C and 70 MPa. Results show that a longer holding time will short the fatigue life of alloy. The authors also come up with the idea that slip bands are related to fatigue damage and that dislocation density will increase if loading stress increases. Zhen Wang et al. [15] study the creep–fatigue properties of superalloy at high temperature with the scanning electron microscope. They set the variables of the experiment as holding time at maximum tensile stress. Results show that the fatigue life will decrease if increase the holding time slightly. There are also many studies that focus on the low cycle fatigue experiment [16], thermal–mechanical fatigue [17], high cycle fatigue experiment [18], crack initial problems [19], and multiaxial fatigue life prediction [20].

There are many studies that focus on the creep–fatigue prediction model related to energy and unified constitutive models. Siyuan Chen et al. [21] study the fatigue mechanism with creep–fatigue experiment and scanning electron microscopy. The objects of study are FGH96 and GH4169 superalloys. Based on the experiment result, the authors come up with a fatigue life prediction model on the basis of the total strain equation. Tianyu Zhang et al. [22] come up with a life prediction model based on the experiment data. This model considering the effect of creep–fatigue interaction and hybrid-controlled loading. They conduct a hybrid-controlled experiment on the P92 steel at the temperature of 625 °C, and the variables of the experiment are the stress range and holding time. Takehiro Shimada et al. [23] come up with a non-unified constitutive model to predict the fatigue life of 316H steel. The model considering the cycle hardening properties and strain range dependence properties is adopted in a finite element model. Results show that the predicted data are accurate when comparing with experiment data. Run-Zi Wang et al. [24] come up with a new modified strain energy model to predict the creep–fatigue life of alloy in high-temperature components. This model considers the effect of mean stress, stress relaxation condition, and creep condition of alloy. Results show good prediction accuracy of the model. Kai-Shang Li et al. [25] come up with a new calculation process to predict the fatigue life on the basis of the crystal plasticity finite element method. This method can express and predict the alloy behavior macroscopically and microscopically. There are many studies that focus on the semi-decoupled model [26], nonlinear fatigue damage accumulation model [27], digital twin model [28], constant life model [29], and dynamic stall model [30].

There are also many studies related to machine learning on creep–fatigue life prediction. Joeun Choi et al. [31] conduct a series of fatigue experiments to investigate the fatigue properties of hyper-elastic material. They adopted six different machine learning algorithms to predict the fatigue life and the deep neural network exhibits the best, the average error is 14.3%. Hongyixi Bao et al. [32] use a support vector machine algorithm to predict the influence of defect parameters on the fatigue life of Ti–6Al–4V alloy. In the training process, they use the cross-validation method to utilize the data sufficiently and quickly. Zhixin Zhan et al. [33] investigate fatigue failure of the additive manufacturing material 316 steel by different machine learning algorithms combined with continuum damage mechanics. The machine learning algorithms are artificial neural network, random forest, and support vector machine. They ultimately compare the prediction accuracy of different algorithms. Dianyin Hu et al. [34] study the crack growth life prediction using the Bayesian method. The posterior distribution of the Bayesian model is quantified by the Monte Carlo method. To solve the uncertainty transfer problems and to speed the calculation time, the authors adopt the Gaussian process regression method. Wangchen Yan et al. [35] study the bridge failure probability problems by forward neural network and Monte Carlo method. After training by a set of data, the model can be applied to predict the failure probability problems accurately and be used to assess the safety of the bridge. There are also many studies related to artificial neural network [36], deep learning [37], convolutional neural network [38], Gaussian process [39], and hybrid neural network [40].

If the diesel engine works for a long time at high speed and large load, especially in the case of thin air, insufficient combustion, and insufficient heat dissipation in the plateau environment, a higher temperature will be always maintained in the cylinder, which leads to the loading of persistent thermal stress and mechanical stress, and the local plastic deformation of the piston [41]. Considering the instability of the diesel engine working state, the low-cycle fatigue–creep interaction may occur at this time. Moreover, due to uneven heat dissipation, heat accumulation, excessive thermal gradient, and other problems caused by the limitation of the geometric structure of inlet and exhaust valve grooves, rings, and other parts in the piston, the fatigue–creep failure is very easy to occur. Relevant research shows that the aluminum alloy material may have creep, oxidation, dynamic strain aging, creep–fatigue, and ratchet characteristics under the out-of-phase thermal–mechanical coupling load, which will further exacerbate the complexity of the material fatigue response, hindering the modeling of material characteristics and establishing fatigue life model. In order to improve the reliability and fatigue life of the whole machine, it is necessary to establish a life prediction model of piston fatigue failure. An accurate and widely applicable fatigue life prediction model requires an accurate understanding of the response of materials under different loads and working conditions. Therefore, based on the results of finite element analysis, the creep–fatigue experiment is conducted for the piston material 2A80 aluminum alloy in this paper, and the fatigue life prediction model of the piston is established according to the test results and relevant methods. The main tasks accomplished are as follows.

First, the temperature field, thermal stress field, and thermal–mechanical coupling stress field of the piston in the actual working process are calculated and analyzed by finite element model. Second, the creep–fatigue experiment is conducted for the piston material 2A80 aluminum alloy in combination with the results of the maximum temperature and maximum stress calculated by the finite element model. Then, two fatigue life prediction models of aluminum alloy materials are proposed on the basis of the experiment results and relevant algorithms, which are the traditional fatigue life prediction model based on energy dissipation criteria and the fatigue life prediction model based on the twin support vector regression machine, respectively. The results show that the latter is more accurate in predicting the fatigue life of piston materials. Finally, the fatigue life of the piston in the actual working process is predicted and studied by using the proposed fatigue life prediction model of piston aluminum alloy materials.

## 2. Simulation of Piston Temperature and Stress Field

The piston of the heavy-duty diesel engine made of 2A80 aluminum alloy is studied in this article. This aluminum alloy is widely used in the piston, cylinder head, and other high-temperature parts of the heavy-duty diesel engine due to its high-temperature strength, no extrusion effect, corrosion resistance, etc. The main chemical compositions of the aluminum alloy are shown in Table S1 in the Supplementary Materials.

### 2.1. Temperature Field Analysis

**Figure 1:**
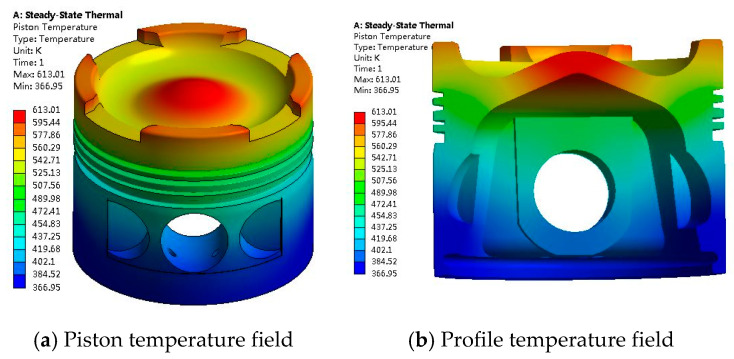
Detailed temperature field of the piston. (**a**) Piston temperature field; (**b**) Profile temperature field.

The three-dimensional geometric model of the piston is established, the boundary conditions such as temperature and pressure in the cylinder are calculated by using the working process model of the diesel engine [42]. The temperature field of the piston is obtained with the finite element method, as shown in Figure 1. The finite element results in this article were obtained by the software of ANSYS 19.0 produced by ANSYS, Inc. (Canonsburg, PA, USA).

As can be seen from Figure 1, from top to bottom, the temperature field of the piston shows a change from high to low, and its maximum temperature is 613.01 K and distributed near the center circle of the piston top. The higher temperature areas also appear in the convex parts besides the inlet and exhaust valve grooves at the piston top. The temperatures of these two parts are high because they directly withstand the erosion of high-temperature gases in the cylinder and are far from the skirt and piston ring, which are the main heat dissipation areas in the piston. Although the piston temperature is high in the local area, its maximum temperature does not exceed 650 K of the aluminum alloy material failure temperature. In addition, the temperature of the piston ring and skirt areas does not exceed the carbonization temperature of 450 K of the lubricating oil.

**Figure 2:**
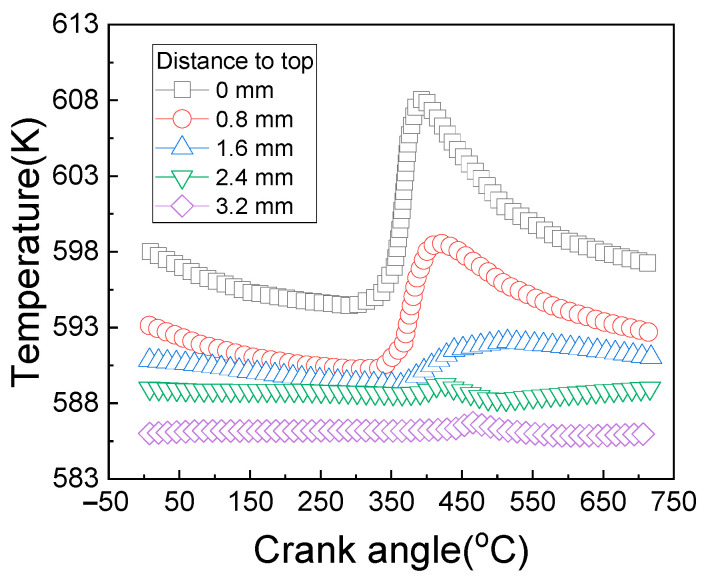
Temperature fluctuations at different depths from the piston top.

Meanwhile, the change rule of the temperature of the monitoring points, which is at different depths from the piston top, with the crankshaft rotation angle in a work cycle of the diesel engine is calculated, as shown in Figure 2.

As can be seen from Figure 2, the influence of the in-cylinder gas fluctuation on the piston temperature is only reflected on the top surface of the piston. With the increase of depth, the temperature fluctuation tends to be gentle. When the depth exceeds 2.4 mm, the temperature basically remains unchanged.

### 2.2. Thermal Stress Field Analysis

**Figure 3:**
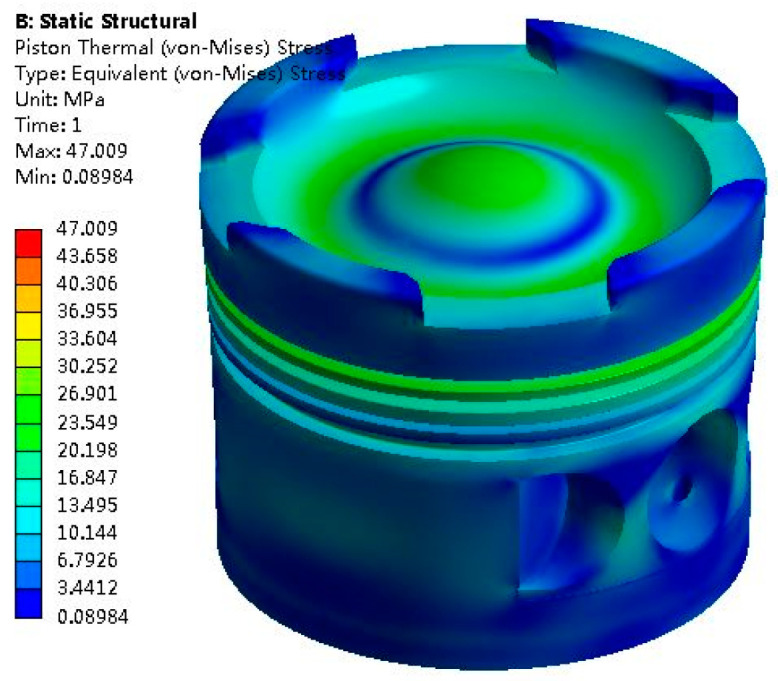
Piston thermal stress field.

The calculated results of the piston thermal stress field are shown in Figure 3.

As can be seen from Figure 3, the maximum thermal stress of the piston is 47.009 MPa and concentrated at the areas of piston top and ring. The change rule of the piston thermal stresses at different positions with the crankshaft rotation angle in a work cycle is shown in Figure S1 in the Supplementary Materials.

As can be seen from Figure S1, in a work cycle, the thermal stress fluctuation on the piston top surface is larger (feature points C, D, and E), and the closer to the center of the top surface, the greater the fluctuation amplitude is. The thermal stresses on the bottom surfaces of the piston top (feature points A and B) almost remain unchanged; this is because the temperature fluctuations tend to be gentle with the increase of depth from top to bottom, and the bottom of piston top’s heat transfer is almost unchangeable, and hence thermal stress of that location tends to be gentle.

### 2.3. Thermal–Mechanical Coupling Stress Field

**Figure 4:**
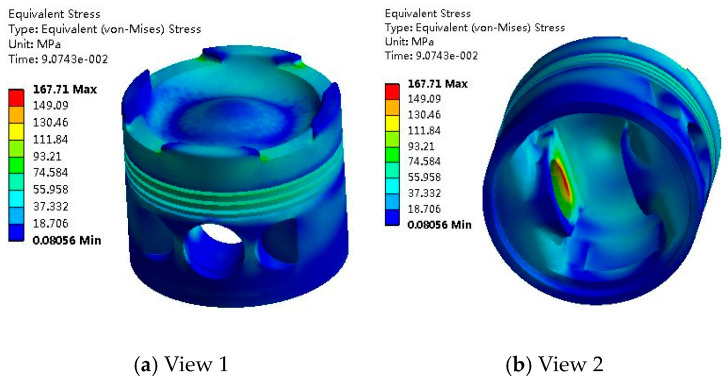
Distribution of piston thermal–mechanical coupling stress field. (**a**) View 1 (**b**) View 2.

The thermal–mechanical coupling stress field of the piston is shown in Figure 4.

As can be seen from Figure 4, the maximum thermo-mechanical coupling stress of the piston is 167.71 MPa and is mainly distributed in the inner and upper areas of the piston pinhole. The piston pin is connected to convert the energy generated by combustion in the cylinder into the kinetic energy for crankshaft rotation, so the force is larger. At the corner of the inlet and exhaust valve groove on the top surface of the piston, the thermo–mechanical coupling stress is also large. This is because the corner is subjected to a large heat load (large heat flow) and a mechanical load (gas burst pressure in the cylinder) and has a stress concentration phenomenon by the chamfering without a smooth transition.

**Figure 5:**
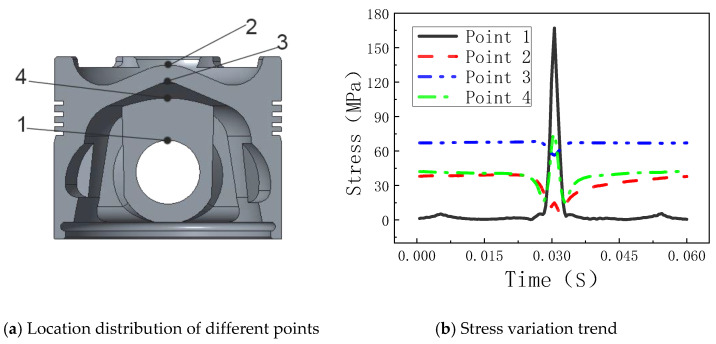
Change rule of the coupling stresses at different points in the piston with time. (**a**) Location distribution of different points; (**b**) Stress variation trend.

Figure 5 shows the change rule of the coupling stresses at different points in the piston with time.

As can be seen from Figure 5, the coupling stress of each point is directly affected by the gas pressure, and the stress changes are most dramatic near the moment of pressure outbreak. The stress change rule of node 1 is very similar to the gas pressure change in the cylinder; this is because the gas pressure directly causes the change of stress at this place. Usually, the greater the gas pressure is, the greater the force exerted on the pin seat is.

Four key points in Figure 5a are selected as study objects, and the equivalent piston stress amplitudes under two working conditions are calculated as shown in Figure S2 in the Supplementary Materials.

As can be seen from Figure S2, the equivalent stress amplitude under the plain conditions is smaller than that of the plateau conditions, and the amplitude of node 2 is larger than that of node 3, which is caused by the high temperature on the piston top surface and the violent fluctuations. The amplitude of node 1 is much greater than that of node 4, but according to the practical experience, the fatigue life of node 4 is also short. On the one hand, this is because the latter’s temperature is above 100 K higher than the former; on the other hand, this is because the stress of node 1 stress in a cycle is basically at the compressive stress state, while node 4 is tensile stress in most of the time, and the tensile stress will accelerate the process of fatigue damage.

### 2.4. Brief Summary

By analyzing the temperature field, thermal stress field, and mechanical stress field of the piston, it can be found that the working environment of the piston is relatively poor, and there is large temperature stress in the local area. If the diesel engine works for a long time under the working conditions of high speed and large load, it is prone to fatigue, creep, and other forms of failures in the piston. Parts that are prone to fatigue failures mainly include the center area of the piston top surface, the corner at the bottom of the inlet and exhaust valve grooves of the top surface, the piston ring groove, and the interior of the piston pin seat.

In order to explore the failure mechanism and fatigue life of the piston, it is necessary to establish the model of damage accumulation and fatigue failure, which should be based on accurate fatigue experiment data. Therefore, the fatigue test of aluminum alloy materials is mainly introduced below.

## 3. Creep–Fatigue Test of Aluminum Alloy Materials

### 3.1. Test Schemes

The creep–fatigue test carried out in this paper is controlled by the strain of the specimen. In combination with the results of the finite element analysis and the actual working conditions of the diesel engine, the determined test variables mainly include the temperature (530 K, 560 K, 590 K, and 620 K), the strain range (0.8%, 0.6%), the strain ratio (0, −1), the creep loading time (0 s, 60 s, 120 s), etc.

The equipment used in the test is the QBR-50 high-temperature durable creep testing machine (Shandong, China), which is mainly used to test the creep properties of various metals and alloy materials in the high-temperature environment and can be used to test and record the creep limit, stress–strain response, cycle numbers, etc. The main parameters of the testing machine are shown in Table S2 in the Supplementary Materials.

Although the piston is continuously eroded by high-temperature and high-pressure gases during operation, it can be considered that the piston is loaded under constant pressure at high temperature due to the high frequency (up to 500 times per minute to 1000 times per minute) of explosion pressure. If a diesel engine works under high speed (higher frequency) and large load (higher temperature and higher pressure) for a long time, the aluminum alloy material of the piston may suffer from creep–fatigue failure due to harsh conditions. In order to reflect the environment of the piston in the actual working process as true as possible, the waveform in the creep–fatigue loading process is set to the style shown in Figure S3 in the Supplementary Materials [43].

As can be seen from Figure S3, the loading waveform of the creep–fatigue test is based on the waveform of the low-cycle fatigue test—when the low-cycle fatigue is loaded to the maximum negative strain, keep this strain for a certain time (60 s or 120 s); then continue to test according to the loading path of the low-cycle fatigue and continue to carry out creep loading until the maximum negative strain of the next cycle is reached.

Combined with the results obtained by the finite element model and the actual operating conditions of the diesel engine, a total of 24 group tests are designed to study the creep–fatigue characteristics of materials. The detailed test conditions are shown in Table S3 in the Supplementary Materials.

### 3.2. Processing of Specimens

As per the requirements of the Metallic materials—Uniaxial creep testing method in tension (GB/T 2039-2012), the specimens are processed and tested, and the specific processing dimensions are shown in Figure S4 in the Supplementary Materials.

### 3.3. Analysis of Test Results

#### 3.3.1. Overview of Results

**Figure 6:**
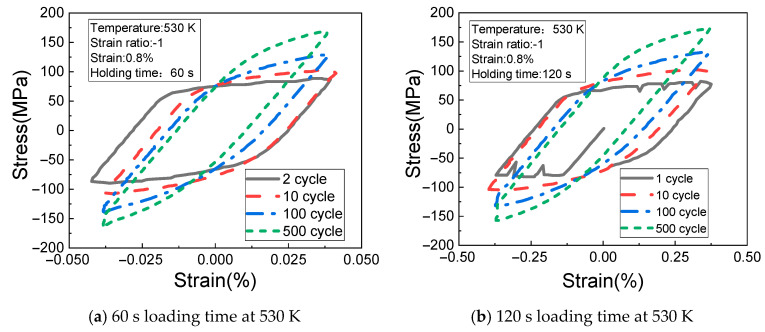
Results of different loading times at 530 K. (**a**) 60 s loading time at 530 K; (**b**) 120 s loading time at 530 K.

Figure 6a,b shows the change rules of the cyclic stress (measured at the temperature of 530 K, the strain of 0.8%, the strain ratio of −1, and the creep load holding time of 60 s and 120 s) with the cyclic strain. It can be seen from the figures that the stress fluctuation occurs in the first cycle of the material when the load holding time is 120 s, and the stress fluctuation gradually disappears with the increase of cycle times. At the same time, the maximum tensile stress and the maximum compressive stress of the material increase with the number of cycles, and the stress relaxation appears at 120 s holding time at 100 cycles.

**Figure 7:**
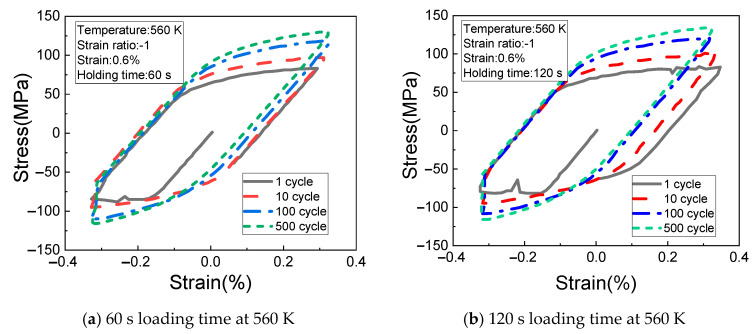
Results of different loading times at 560 K. (**a**) 60 s loading time at 560 K; (**b**) 120 s loading time at 560 K.

Figure 7a,b shows the change rules of the cyclic stress (measured at the temperature of 560 K, the strain of 0.6%, the strain ratio of −1, and the creep loading time of 60 s and 120 s) with the cyclic strain. It can be seen from the figures that the stress fluctuation of the material in the first cycle still does not disappear, and the stress relaxation is gradually obvious.

Figure S5a,b in the Supplementary Materials show the change rules of the cyclic stress (measured at the temperature of 590 K, the strain of 0.5%, the strain ratio of −1, and the creep loading time of 60 s and 120 s) with the cyclic strain. It can be seen from the figures that the material has viscous properties at higher temperatures, and the stress relaxation amplitude gradually increases with the increase of cycle numbers.

Figure S6a,b in the Supplementary Materials show the change rules of the cyclic stress (measured at the temperature of 620 K, the strain of 0.4%, the strain ratio of −1, and the creep loading time of 60 s and 120 s) with the cyclic strain. Different from the above test results, although the material has stress relaxation in the first cycle, the maximum tensile stress of the material does not change with the increase of cycle numbers. When the 500th cycle is reached, the maximum compressive stress and the stress relaxation amplitude of the material decrease when the holding time is 60 s.

#### 3.3.2. Creep–Fatigue Stress Relaxation

**Figure 8:**
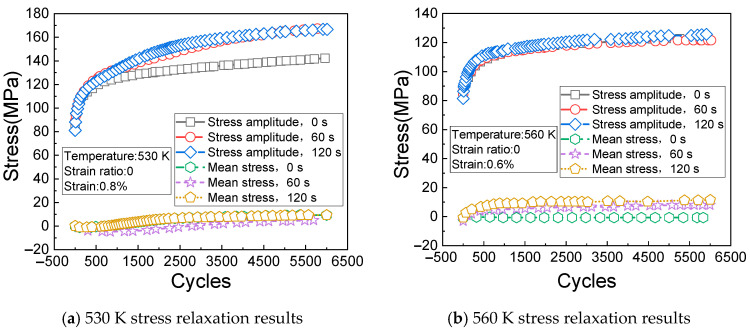
Stress relaxation results at 530 K and 560 K. (**a**) 530 K stress relaxation results; (**b**) 560 K stress relaxation results.

Figure 8a shows the change rules of the cyclic stress (measured at the temperature of 530 K, the strain of 0.8%, and the strain ratio of 0) with the times of cycles. It can be seen from Figure 8a that the stress relaxation caused by creep gradually appears after 500 cycles. Figure 8b shows the change rules of the cyclic stress (measured at the temperature of 560 K, the strain of 0.6%, and the strain ratio of 0) with the times of cycles.

**Figure 9:**
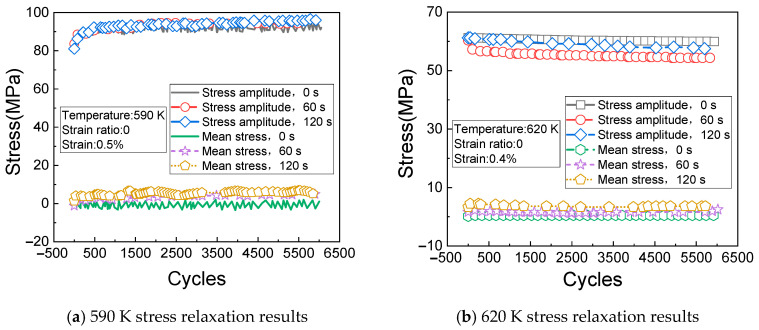
Stress relaxation results at 590 K and 620 K. (**a**) 590 K stress relaxation results; (**b**) 620 K stress relaxation results.

Figure 9a shows the change rules of the cyclic stress (measured at the temperature of 590 K, the strain of 0.5%, and the strain ratio of 0) with the times of cycles. It can be seen from Figure 9a that the cyclic response rule of the material is very unstable under the current test conditions, but the overall change trend is increasing and then stabilizing with the increase of cycles.

Figure 9b shows the change rules of the cyclic stress (measured at the temperature of 620 K, the strain of 0.4%, and the strain ratio of 0) with the times of cycles. It can be seen from Figure 9b that the stress amplitude of the material gradually decreases with the increase of creep loading time.

**Figure 10:**
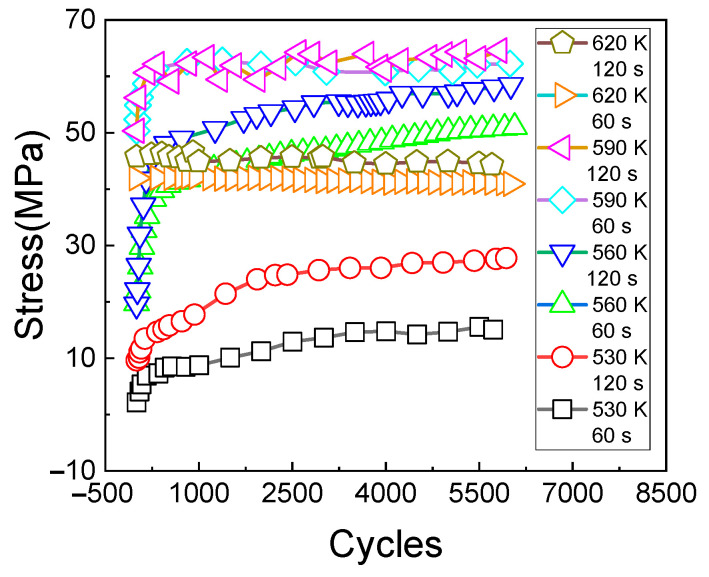
Stress changes with cycles.

By summarizing the test results under different conditions, the change rule of stress relaxation values with the cycles can be obtained, as shown in Figure 10. It can be seen from Figure 10 that, regardless of the influence of temperature and loading time, the relaxation stress of the material gradually increases with the increase of cycles and finally tends to be stable. However, these test results are obtained at different temperatures, and the comparison between the absolute values is not convincing. Therefore, the concept of equivalent relaxation stress is introduced to analyze the results further.

The calculation formula of equivalent relaxation stress is as Equation (1):

(1) $$\sigma_{e}=\frac{\sigma }{\sigma_{max}}$$

where $\sigma_{e}$ indicates the equivalent relaxation stress, σ indicates the original relaxation stress, and $\sigma_{max}$ indicates the maximum cyclic response stress.

**Figure 11:**
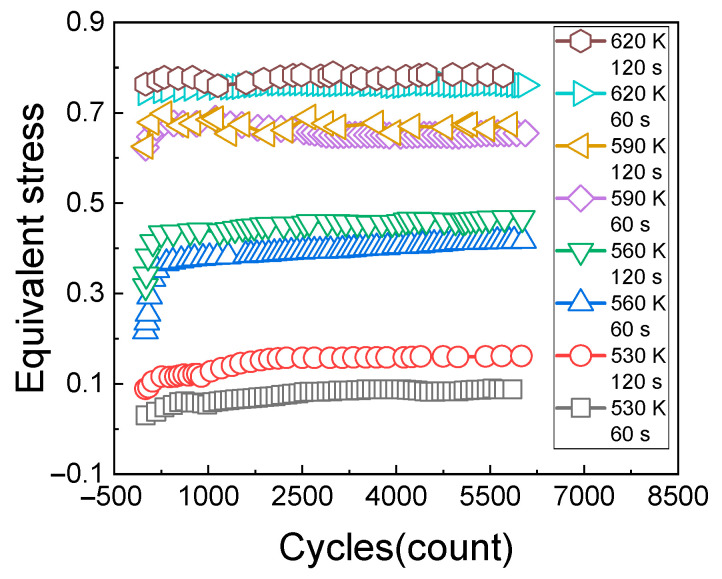
Equivalent stress changes with cycles.

Under the conditions of different temperatures and loading times, the change rule of the equivalent relaxation stress with the cyclic times is shown in Figure 11. It can be seen from Figure 11 that, different from Figure 10 in which the relaxation stress at a higher temperature (620 K) is less than that at a lower temperature (590 K), the equivalent relaxation stress increases with the increase of temperature, which shows that the material viscosity increases with the increase of temperature. It can also be seen from Figure 10 that the longer the creep holding time is, the greater the stress relaxation amplitude is.

#### 3.3.3. Impacts of Holding Time

**Figure 12:**
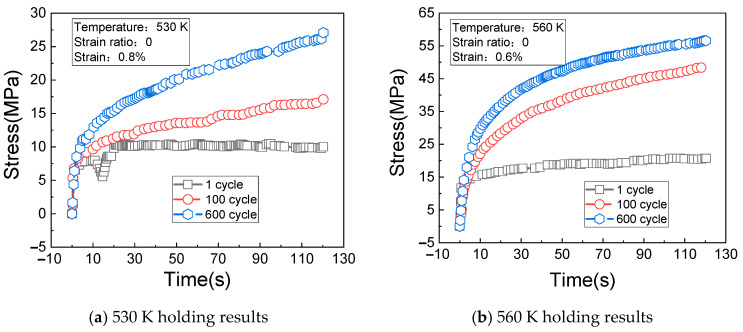
Stress relaxation results of holding at 530 K and 560 K. (**a**) 530 K holding results; (**b**) 560 K holding results.

Figure 12a shows the change rules of the cyclic stress (measured at the temperature of 530 K, the strain of 0.8%, the strain ratio of 0, and the creep loading time of 120 s) with the holding time. Figure 12b shows the change rules of the cyclic stress (measured at the temperature of 560 K, the strain of 0.6%, the strain ratio of 0, and the creep loading time of 120 s) with the holding time.

**Figure 13:**
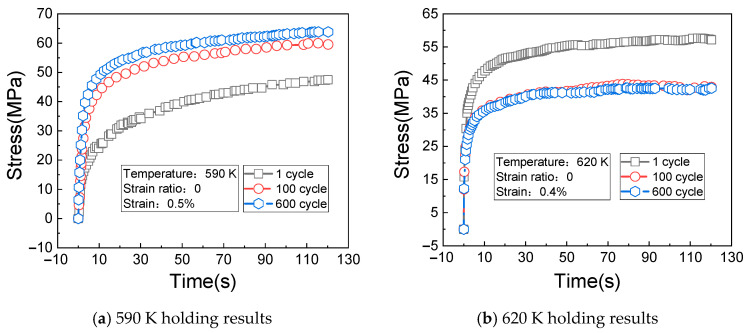
Stress relaxation results of holding at 590 K and 620 K. (**a**) 590 K holding results; (**b**) 620 K holding results.

Figure 13a shows the change rules of the cyclic stress (measured at the temperature of 590 K, the strain of 0.5%, the strain ratio of 0, and the creep loading time of 120 s) with the holding time. Figure 13b shows the change rules of the cyclic stress (measured at the temperature of 620 K, the strain of 0.4%, the strain ratio of 0, and the creep loading time of 120 s) with the holding time.

It can be seen from Figure 13a,b that the stress relaxation amplitude of the material increases with the increase of cycles and holding time; under the test conditions in which the temperature is 620 K and the strain is 0.4%, the stress of the first cycle is larger. When the 600th cycle is reached, the response stress of the material tends to be stable with the extension of the holding time, indicating that stress saturation occurs at this time.

**Figure 14:**
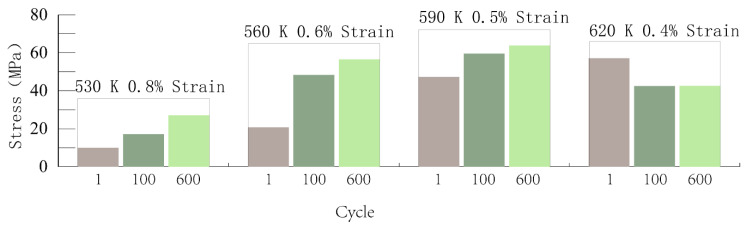
Summary of relaxation stresses under different conditions.

The maximum relaxation stresses corresponding to three different cycles in four temperature conditions at the holding time of 120 s are summarized, and the results are shown in Figure 14.

Combined with the above analysis and figures, it can be found that the impacts of the temperature and strain range on the material stress relaxation are very significant.

### 3.4. Brief Summary

The following conclusions can be obtained by synthesizing the results under different test conditions:

1. Under the creep–fatigue loading conditions, a certain degree of stress relaxation occurs in all temperature ranges. With the increase of temperature, the stress relaxation amplitude of material increases obviously, which indicates the important impact of temperature conditions on the creep stress relaxation;
2. At lower temperatures, the material experiences the phenomenon of cyclic hardening and stress relaxation. With the increase of temperature, the degree of cyclic hardening decreases obviously, but the stress relaxation amplitude increases gradually;
3. With the increase of temperature and the extension of holding time, the cyclic stress response of the material increases gradually.

## 4. Fatigue Life Prediction Models of Aluminum Alloy Materials

Based on the creep–fatigue test results and preliminary conclusions of aluminum alloy materials, two models are established to predict the fatigue life of the piston, which refer to the creep–fatigue life prediction model based on energy dissipation criteria [44] (EDC model) and the creep–fatigue life prediction model based on the nonlinear least-square twin support vector regression machine algorithm [45] (NLTS algorithm), respectively. The EDC model is mainly based on the energy method to quantify the damage and impact of the load and used to obtain the fatigue life of the piston, and the NLTS algorithm is mainly used to establish the relationship between the loading conditions and the fatigue life with the machine learning method based on the test results and predict the piston life in the actual working process.

### 4.1. Creep Fatigue Life Prediction Model Based on Energy Dissipation Criteria

#### 4.1.1. Overview

In essence, the energy-based fatigue life prediction method is to match the fatigue load with some form of energy, and then to use this energy to quantify the damage degree of the load, so as to evaluate the fatigue life of the material. From the microscopic point of view, the damage caused by each cyclic load to the material can be regarded as that the energy carried by the load provides the power to move the internal phase structure of the material. With the accumulation of time and load, the phase movement eventually leads to qualitative changes (macroscopic damage) from quantitative changes (phase movement) and causes the fatigue failure of the material. With the deepening of theoretical research and the development of various hardware and software facilities, scholars have proposed many energy-based methods for predicting the fatigue life, and these fatigue failure criteria can be expressed as Equation (2).

(2) $$\sum_{i=1}^{N_{f}}\Delta W_{i}=W_{f}^{\prime },$$

where $N_{f}$ indicates the number of loads that causes the fatigue failure of the material, $\Delta W_{i}$ indicates the damage caused by the ith cycle of loads to the material, and $W_{f}^{\prime }$ indicates the damage critical quantity of fatigue failure of the material. Based on different directions and emphases, different types of energies can be determined to evaluate the damage of the cyclic load, mainly including the total strain energy, the cyclic hysteresis energy, and the partial cyclic hysteresis energy. Two problems shall be solved in this process—the first problem is to determine what kind of energy plays a leading role in its life, and the second is to find out the mathematical relationship between the energy and the fatigue life.

#### 4.1.2. Cyclic Hysteresis Energy of Low Cycle Fatigue

According to the basic theory, the strain energy (elastic and plastic strain energy) corresponding to the cyclic response of the material under low-cycle fatigue loads is the most important failure energy, and there is a linear relationship between the strain energy and its fatigue life, which is Equation (3).

(3) $$W=C+DN_{f},$$

where W indicates the cyclic response strain energy of the material, and C and D indicate the constants related to the material properties. The material will produce large plastic deformation when subjected to low-cycle fatigue loads, and the plastic strain energy is the main energy input by the external load to the material, but most of it is converted into heat and disappeared to the environment. The plastic strain energy input by each cycle is $\Delta W_{p}$ and measured based on the area of the hysteresis loop (therefore, it is also called cyclic hysteresis energy). If the energy can be linearly superimposed, the total energy absorbed by the material before its failure is the sum of the areas of all cyclic hysteretic loops. Relevant studies show that in a stable low-cycle fatigue test process, when the load is relatively stable, that is, the $\Delta W_{p}$ generated by per cycle is basically constant, the relationship between the cyclic hysteresis energy and the low-cycle fatigue life can be expressed by the Equation (4):

(4) $$\Delta W_{p}=kN_{f}^{a}+c$$

where k, a and c are constants related to material properties.

For the material with mashing characteristics (the hysteresis curve is symmetric to the origin), the expression of its cyclic plastic hysteresis energy is Equation (5):

(5) $$\Delta W_{p}=\frac{1-n^{\prime }}{1+n^{\prime }}\Delta \sigma \Delta \varepsilon_{p,}$$

where Δσ indicates the range of the cyclic stress, $\Delta \varepsilon_{p}$ indicates the plastic strain range of cyclic response, and $n^{\prime }$ indicates the cyclic hardening index.

For the material without mashing characteristics (the hysteresis curve is asymmetric to the origin), the expression of its cyclic plastic hysteresis energy is Equations (6) and (7):

(6) $$\Delta W_{p}=\frac{1-n^{\prime }}{1+n^{\prime }}\left(\Delta \sigma -\delta_{\sigma }\right)\Delta \varepsilon_{p}+\delta_{\sigma }\Delta \varepsilon_{p}$$

(7) $$\delta_{\sigma }=\Delta \sigma -2E{\left(\frac{\Delta \varepsilon_{p}}{2}\right)}^{n^{\prime }},$$

where E indicates the elastic modulus of the material.

During fatigue loading, not all hysteresis energy of cyclic response is absorbed by the material and used to cause damages; a large part of the energy is dissipated through acoustic emission and thermal radiation. That is to say, only part of the cyclic hysteresis energy is used to generate dislocation in the material and help the growth and expansion of micro-cracks. This part of the energy acting on damage is called the fatigue damage effective energy and is expressed by $W_{e}$. This energy is only a part of the cyclic plastic hysteresis energy during fatigue loading, and can be expressed by the mathematical formula as Equation (8):

(8) $$W_{e}=\sum \alpha \Delta W_{p}$$

where α indicates the coefficient relating to material properties, generally between 0 and 1, and can be determined according to the test data.

In general, with the increase of the fatigue load (plastic deformation) at a constant temperature, the total hysteresis energy absorbed by the material decreases gradually. However, under the same fatigue load, the total hysteresis energy absorbed by the material increases with the increase of temperature. The plastic strain amplitude of load is the main factor for determining the fatigue life of the material. As a result, with the increase of the plastic strain amplitude, the proportion of the fatigue damage effective energy in the total hysteresis energy in each load cycle will also increase. Therefore, in the process of low-cycle fatigue failure, the amount of the hysteresis energy converted into the effective fatigue damage energy is positively correlated with the plastic strain amplitude. Relevant studies show that the proportion of the hysteresis energy converted to the fatigue damage energy in each cycle is the power function of the ratio of the plastic strain amplitude to the total strain amplitude, which is Equation (9).

(9) $$\alpha_{i}={\left(\frac{\Delta \varepsilon_{pi}}{\Delta \varepsilon_{i}}\right)}^{m},$$

where $\Delta \varepsilon_{i}$ indicates the total strain amplitude of the ith cycle, $\Delta \varepsilon_{pi}$ indicates the plastic strain amplitude of the ith cycle, and m indicates the energy conversion index.

The effective fatigue damage energy converted in each cycle is Equation (10).

(10) $$W_{ci}=\alpha_{i}\Delta W_{pi}={\left(\frac{\Delta \varepsilon_{pi}}{\Delta \varepsilon_{i}}\right)}^{m}\Delta W_{pi},$$

where $W_{ci}$ indicates the effective fatigue damage energy of the material in the ith cycle.

Combined with the Equations (10) and (4), the Equation (11) is obtained:

(11) $${\left(\frac{\Delta \varepsilon_{pi}}{\Delta \varepsilon_{i}}\right)}^{m}\Delta W_{pi}=kN_{f}^{a}+c.$$

#### 4.1.3. Cyclic Hysteresis Energy of Creep–Fatigue

Under the condition of creep–fatigue deformation of the material, the area of its hysteresis curve will change. This impact should be considered during the calculation and analysis of the fatigue life through the cyclic hysteresis energy. Assuming that the plastic strain energy caused by creep is $\Delta W_{creep}$, then:

(12) $$\Delta W_{creep}=\Delta \sigma *\varepsilon_{creep},$$

where $\varepsilon_{creep}$ indicates the creep deformation of the material under creep loading. According to the low-cycle fatigue, the effective damage energy under the low-cycle creep composite loading conditions (which is closest to the actual working state of the material at this time) is Equation (13):

(13) $$W_{ci}=\alpha_{i}\left(\Delta W_{pi}+\Delta W_{creep}\right)={\left(\frac{\Delta \varepsilon_{pi}}{\Delta \varepsilon_{i}}\right)}^{m}\left(\Delta W_{pi}+\Delta W_{creep}\right).$$

Therefore, the formula of fatigue life is Equation (14):

(14) $${\left(\frac{\Delta \varepsilon_{pi}}{\Delta \varepsilon_{i}}\right)}^{m}\left(\Delta W_{pi}+\Delta W_{creep}\right)=kN_{f}^{a}+c.$$

All parameters are calculated with the above formula and test data, and the values of each parameter are obtained as shown in Table S4 in the Supplementary Materials.

### 4.2. Creep–Fatigue Life Prediction Model Based on the Twin Support Vector Machine

The creep–fatigue life prediction model of aluminum alloy materials is mainly established according to the energy dissipation criteria above, but this model has some problems; for example, there are too many simplifications and assumptions in the establishment process, the obtained model parameters cannot be dynamically adjusted with the results of subsequent tests. In order to compare and verify the above models and considering the breakthrough of the machine learning algorithm in this field, the creep–fatigue life of aluminum alloy materials is mainly predicted based on the nonlinear least-square twin support vector regression machine algorithm (NLTS algorithm), which is described in detail below.

#### 4.2.1. Support Vector Machine

The support vector machine [46] (SVM) method is proposed by former Soviet scientist Vapnik and is a machine learning algorithm that can use small sample data to solve classification and regression problems. If $\left(x_{i},y_{i}\right)$ is the train data and the number of samples is n, then:

(15) f(x)=ωφ(x)+b,

where x represents the vector of the sample, ω represents the weight vector, and b represents the offset vector, which is also commonly known as the classification threshold.

If there is a plane that can separate the different samples in the geometric space,

(16) f(x)=0.

Thus, the Equation (17) is valid:

(17) $$y_{i}*f\left(x\right)=y_{i}\left[\omega \varphi \left(x_{i}\right)+b\right]>0.$$

Then, f(x) is deemed as a hyperplane. Considering the arbitrariness of the offset vector b, the above hyperplane solution can be transformed into a solution of a convex optimization problem,

(18) $$\min:\frac{1}{2}{∥\omega ∥}^{2}$$

(19) $$s.t.y_{i}\left(\omega^{T}x_{i}+b\right)\geq 1,$$

where $\frac{1}{∥\omega ∥}$ is the distance between two support vectors, the process of evaluating its maximum value is transformed into the goal of evaluating the minimum value of ${∥\omega ∥}^{2}$ in the convex optimization problem, and the coefficient $\frac{1}{2}$ is used to eliminate the coefficient after derivation of ${∥\omega ∥}^{2}$, which is of no engineering meaning.

The Lagrange function is also introduced as Equation (20):

(20) $$L\left(\omega ,b,a\right)=\frac{1}{2}{∥\omega ∥}^{2}-\overset{l}{\underset{i=1}{\sum }}a_{i}\left[y_{i}\left(\omega x_{i}+b\right)-1\right],$$

where $a_{i}$ indicates the Lagrange multiplier, which is a number that is constantly greater than zero.

The above problem is further transformed into its dual problem, that is, the differential coefficient of Lagrange function to ω and b is zero at the solution of the problem, and therefore,

(21) $$\max:\overset{l}{\underset{j=1}{\sum }}a_{j}-\frac{1}{2}\overset{l}{\underset{i=1}{\sum }}\overset{l}{\underset{j=1}{\sum }}a_{i}a_{j}y_{i}y_{j}\left(x_{i}x_{j}\right)$$

(22) $$s.t.\;\overset{l}{\underset{j=1}{\sum }}a_{j}y_{j}=0$$

(23) $$a_{j}\geq 0.$$

The optimal $a^{*}$, the optimal weight vector $\omega^{*}$, and the optimal offset vector $b^{*}$ can be solved in the form of the Equations (24)–(26):

(24) $$a^{*}={\left(a_{1}^{*},a_{2}^{*},\cdots ,a_{l}^{*}\right)}^{T}$$

(25) $$\omega^{*}=\overset{l}{\underset{j=1}{\sum }}a_{j}^{*}y_{j}x_{j}$$

(26) $$b^{*}=y_{i}-\overset{l}{\underset{j=1}{\sum }}a_{j}^{*}y_{j}\left(x_{j}x_{i}\right).$$

At this time, the hyperplane is Equation (27).

(27) $$\omega^{*}x+b^{*}=0.$$

In order to improve the generalization ability of the algorithm, the relaxation variable $\xi_{i}$ is generally introduced into the constraints, and then

(28) $$\min:\frac{1}{2}{∥\omega ∥}^{2}+c\overset{n}{\underset{i=1}{\sum }}\xi_{i}$$

(29) $$s.t.y_{i}\left(\omega^{T}x_{i}+b\right)\geq 1-\xi_{i}$$

(30) $$\xi_{i}\geq 0,$$

where c indicates the penalty factor.

According to the Mercer theorem, the sufficient and necessary condition for a function to be an inner product operation in the feature space is Equation (31).

(31) $$\int \int K\left(x,x^{\prime }\right)\varphi \left(x\right)\varphi \left(x^{\prime }\right)dxdx^{\prime }>0.$$

Therefore, the problem that the dimension of φ(x) in space is too high to be solved easily can be solved through the kernel function $K\left(x,x^{\prime }\right)$.

In this paper, the Gaussian radial basis function is selected as the kernel function:

(32) $$K\left(x_{i},x\right)=exp\left(-\frac{{∥x_{i}-x_{j}∥}^{2}}{\sigma^{2}}\right).$$

#### 4.2.2. Nonlinear Support Vector Regression Machine

The nonlinear support vector regression machine can be expressed as the following convex optimization problems:

(33) $$\min\;\frac{1}{2}{∥\omega ∥}^{2}+ce^{T}\left(\xi +\xi^{*}\right)$$

(34) s.t.Y−[φ(A)ω+be]≤εe+ξ

(35) $$\left[\varphi \left(A\right)\omega +be\right]-Y\leq \varepsilon e+\xi^{*}$$

(36) ξ≥0

(37) $$\xi^{*}\geq 0.$$

At this time, the dual optimization problem is transformed into

(38) $$\min\;\varepsilon e^{T}\left(\alpha +\alpha^{*}\right)-Y^{T}\left(\alpha -\alpha^{*}\right)+\frac{1}{2}{\left(\alpha -\alpha^{*}\right)}^{T}K\left(A,A^{T}\right)\left(\alpha -\alpha^{*}\right)$$

(39) $$s.t.\;e^{T}\left(\alpha +\alpha^{*}\right)=0$$

(40) 0≤α≤Ce

(41) $$0\leq \alpha^{*}\leq Ce,$$

where $\alpha^{*}$ indicates the Lagrange multiplier, and e indicates the unit column vector.

#### 4.2.3. Nonlinear Least-Square Twin Support Vector Regression Machine

The basic idea of the twin support vector machine [47] is to determine a set of functions on both sides of the training sample data to constrain the insensitive upper and lower boundaries of support vector regression.

The insensitive lower boundary is set as Equation (42):

(42) $$f_{1}\left(x\right)=K\left(x^{T},A^{T}\right)\omega_{1}+b_{1}.$$

The insensitive upper boundary is set as Equation (43):

(43) $$f_{2}\left(x\right)=K\left(x^{T},A^{T}\right)\omega_{2}+b_{2}.$$

At this time, the optimization objectives and constraints are changed as Equations (44)–(47):

(44) $$\min\;\frac{1}{2}{∥Y-e\varepsilon_{1}-\left[K\left(A,A^{T}\right)\omega_{1}+eb_{1}\right]∥}^{2}+\frac{1}{2}c_{1}\xi^{T}\xi$$

(45) $$s.t.\;Y-\left[K\left(A,A^{T}\right)\omega_{1}+eb_{1}\right]=e\varepsilon_{1}-\xi$$

(46) $$\min\;\frac{1}{2}{∥Y-e\varepsilon_{2}-\left[K\left(A,A^{T}\right)\omega_{2}+eb_{2}\right]∥}^{2}+\frac{1}{2}c_{2}\eta^{T}\eta$$

(47) $$s.t.\;\left[K\left(A,A^{T}\right)\omega_{2}+eb_{2}\right]-Y=e\varepsilon_{2}-\eta .$$

ω and b can be obtained using

(48) $$\left[\begin{array}{c} \omega_{1}\\ b_{1} \end{array}\right]=\left(1+\frac{1}{c_{1}}\right){\left(E^{T}E+\frac{1}{c_{1}}E^{T}E+𝓌I\right)}^{-1}Ef$$

(49) $$\left[\begin{array}{c} \omega_{2}\\ b_{2} \end{array}\right]=\left(1+\frac{1}{c_{2}}\right){\left(E^{T}E+\frac{1}{c_{2}}E^{T}E+𝓌I\right)}^{-1}Ef,$$

where 𝓌 is the regularization parameter which is usually a positive number close to zero.

#### 4.2.4. Data Normalization and Cross-Validation Methods

The test conditions and the corresponding creep–fatigue life are shown in Table S5 in the Supplementary Materials.

Before training, normalize the data using the Equation (50):

(50) $$x^{\prime }=\frac{x-mean\left(x\right)}{std\left(x\right)}$$

where $x^{\prime }$ is the normalized data, x is the original data, mean(x) is the mean of the same type of data, and std(x) is the standard deviation of the same type of data.

In order to make full use of the test data, the S-fold cross-validation method [48] is used for training and verification. In this method, the data are randomly divided into S parts, one of S−1 part is selected for training, the remaining one part is used to verify, and this process can be repeated for S times; finally, the optimal regression model is determined by considering the training error and the test error. In this paper, the above 24 groups of data are divided into six parts for training and testing, that is, the S value is 6.

### 4.3. Result Analysis

The indexes root mean square error (RMSE) and $R_{a}^{2}$ to evaluate two fatigue life prediction methods are defined in this section. The index RMSE is defined as Equation (51).

(51) $$\mathrm{RMSE}=\sqrt{\frac{1}{m}\overset{m}{\underset{i=1}{\sum }}{\left(y_{i}-\hat{y_{i}}\right)}^{2},}$$

where m indicates the number of samples, $y_{i}$ indicates the creep–fatigue life value obtained from the test, and $\hat{y_{i}}$ indicates the creep–fatigue life value obtained from prediction.

The index $R_{a}^{2}$ is defined as Equation (52).

(52) $$R_{a}^{2}=1-\frac{\left(1-R^{2}\right)\left(m-1\right)}{m-p+1}$$

(53) $$R^{2}=1-\frac{\sum {\left(y_{i}-\hat{y_{i}}\right)}^{2}}{\sum {\left[y_{i}-mean\left(y\right)\right]}^{2}},$$

where p indicates the number of features in the sample (four in this paper), and mean(y) indicates the average creep–fatigue life obtained from the test. $\sum {\left[y_{i}-mean\left(y\right)\right]}^{2}$ represents a discrete degree between the original training data and can be used as the denominator to eliminate the effects of differences in the original data on evaluation indexes.

The evaluation results calculated by the NLTS algorithm are further summarized and compared with the evaluation results calculated by the EDC model, and the results are shown in Table S6 in the Supplementary Materials. In the table, the first six groups of data are calculated by the NLTS algorithm, and the seventh group of data is calculated with the EDC model.

Table S7 in the Supplementary Materials can be obtained by normalizing the data of the same column in Table S6 and averaging the index of the same row.

**Figure 15:**
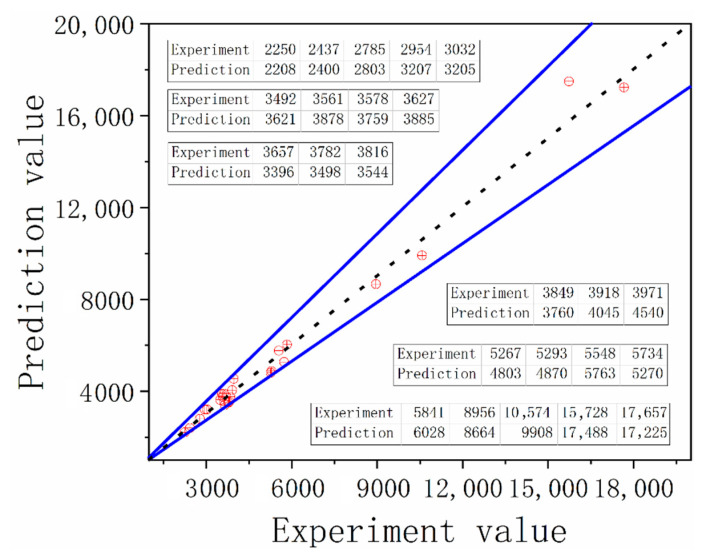
Comparison of the test value of creep–fatigue experiment with the predicted value.

The smaller the index RMSE is, the better it will be, and the rule is the same as to the larger index $R_{a}^{2}$. Considering the values of both indexes according to the results of Table S7, it can be found that the models obtained by the fourth group of training data are optimal. Hence, it is proved that the prediction accuracy based on the NLTS algorithm is better than the method based on the EDC model. By comparing the test value with the predicted value, Figure 15 can be obtained.

## 5. Fatigue Life Prediction Analysis of Piston

**Figure 16:**
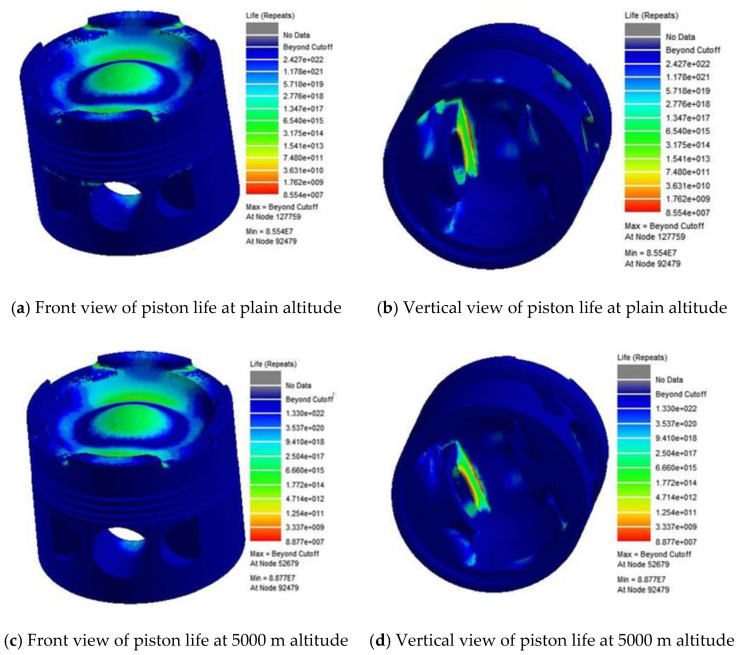
Nephogram of piston fatigue life at different altitudes.

The piston fatigue life under 5000 m altitude and plain conditions is calculated and analyzed using the fatigue life prediction method based on the NLTS algorithm, combined with the piston finite element model, and the results are shown in Figure 16.

The following observations are derived from the figures:

(1) The fatigue hazard points at two altitudes are located in the upper inner part of the piston pin seat. Through the previous analysis, the stress value of this area is largest at the moment of the maximum explosion pressure;

(2) The other fatigue hazard position of the piston is located at the joint between the piston pin seat and the piston top surface; the stress level and the stress amplitude are the second maxima only below the piston pin seat, so the fatigue life is short;

(3) The fatigue life at the inlet and exhaust valve grooves on the top surface of the piston is relatively short, which is mainly caused by the following three aspects: (a) This place is in direct contact with the gas in the cylinder, and the high-temperature environment leads to the smaller fatigue strength of the material; (b) the periodic change of surface temperature and the direct effect of gas explosion pressure lead to significant stress fluctuations there; (c) due to the abrupt geometric structure change of the inlet and exhaust valve grooves, stress concentration is easily generated, which leads to low fatigue life.

(4) The stress level of the bottom surface of the piston top is always greater than that of the top surface, but the fatigue life of the bottom surface is much higher than that of the top surface. It is because the top surface of the piston is directly subjected to high-temperature and high-pressure gases, and the stress fluctuation is more intense. Although the stress value of the bottom surface is large, the stress fluctuation is relatively gentle and the heat load is small; hence, the fatigue life is higher.

According to the fatigue life of piston and the working state of diesel engine, the minimum fatigue lives of the piston under different altitude conditions are 1425.7 h (5000 m altitude) and 2113.6 h (plain condition).

## 6. Conclusions

In this paper, the fatigue life of the piston is predicted and studied by using relevant methods and tests. The study mainly includes the following conclusions:

1. The temperature field, thermal stress field, and thermo–mechanical coupling stress of the piston are calculated and analyzed with the finite element method; results show that the piston highest temperature is 613.01 K, the maximum thermal stress is 47.009 MPa, the maximum thermo-mechanical coupling stress is 167.71 MPa, and the areas with heavy thermal and mechanical loads are mainly concentrated in the center of the top piston, the inlet and exhaust valve grooves, piston pin seat, etc. If the diesel engine works for a long time under the working conditions of high speed and large load, the creep–fatigue failure will occur easily;
2. Based on the finite element calculation results, the creep–fatigue test is carried out for the material 2A80 aluminum alloy of the piston, and the results show that the influence of temperature conditions on creep stress relaxation is very significant. With the increase of temperature, the degree of cyclic hardening decreases gradually, but the stress relaxation amplitude increases. With the increase of temperature and the extension of holding time, the cyclic stress of the material increases gradually;
3. According to the above simulation and test results, the fatigue life prediction models of aluminum alloy materials are proposed with two methods—the creep–fatigue life prediction model based on energy dissipation criteria and the fatigue life prediction model based on twin support vector regression machine. These two methods are verified based on the test results, and the results show that the fatigue life prediction model based on the twin support vector regression machine is more accurate in predicting the life of the creep–fatigue on aluminum alloy materials;
4. The fatigue life prediction model based on the twin support vector regression machine is used to predict the piston fatigue life under the actual service conditions, and the results show that the minimum fatigue life of the piston is 2113.6 h in the plain conditions, and is 1425.7 h at the altitude of 5000 m;
5. Limitations of the research: Firstly, the conditions of the experiment in this research were limited because of our limited budget, and hence we could not obtain the explicit response characteristics of the alloy. Secondly, the study of creep–fatigue prediction model based on the energy dissipation criteria was not enough, and there was still room to improve its prediction accuracy and computational speed. Last but not least, there was no prediction verification for the fatigue life of piston as a result of the limitation of budget and experiment technology;
6. Future work: Firstly, more experiments focused on the response characteristic of alloy should be conducted in the temperature range of 470–650 K, the strain range of 0.3–1.5%, and the loading speed of 0.2–20 cpm. Secondly, the study for creep–fatigue prediction model based on the energy dissipation criteria should receive more attention to investigate the core parameters in predicting the fatigue life of alloy. Lastly, the verification experiment for the actual fatigue life should be conducted if conditions such as the budget and experiment technology allow.

The study in this paper has important guiding significance for the rational use of vehicles in plateau environments, the optimization and improvement of high-temperature components, and the exploration of the properties of aluminum alloy materials.

## Data Availability

All data used in this study can be accessed by the table and figure in the article.

## Supplementary Materials

The following are available online at https://www.mdpi.com/1996-1944/14/6/1403/s1, Table S1. Main chemical compositions of the 2A80 aluminum alloy; Table S2. Main parameters of the high-temperature creep testing machine; Table S3. Summary of creep test conditions; Table S4. Values of each parameter; Table S5. Specific test conditions and results; Table S6. Comparison of training results in different groups; Table S7. Further treatment of evaluation indexes; Figure S1. Change rule of thermal stresses at different positions with the crankshaft rotation angle; Figure S2. Piston equivalent stress amplitudes at different nodes; Figure S3. Loading waveform schematic diagram; Figure S4. Processing requirements of specimens; Figure S5. Results of different loading time at 590 K; Figure S6. Results of different loading time at 620 K.
