# Supplementary material for: Creep–Fatigue Experiment and Life Prediction Study of Piston 2A80 Aluminum Alloy
Source: Materials (Basel). 2021 Mar 13;14(6):1403. doi: 10.3390/ma14061403 (PMC8001697; doi:10.3390/ma14061403)
Supplement: Supplementary file 1 [file materials-14-01403-s001.pdf]

Table S1. Main chemical compositions of the 2A80 aluminum alloy.

**Chemical compositions**	**Si**	**Fe**	**Cu**	**Mn**	**Mg**
Content	0.50-1.20	1.00-1.60	1.90-2.50	0.20	1.40-1.80
**Chemical compositions**	**Zn**	**Ti**	**Ni**	**Al**	**Other**
Content	0.30	0.15	0.90-1.50	margin	0.05

Table S2. Main parameters of the high-temperature creep testing machine.

**Parameters**	**Values**
Maximum experiment force	10kN
Minimum experiment force	1000N
Resolution of experiment force	1/100000
Accuracy of experiment force	≥0.5%
Accuracy of time	±0.1%
Type of furnace	The barrel type furnace
Range of temperature	400-1100K
Length of steady temperature	200mm
Size of inner furnace	φ90mm
Error of temperature	420K-870K，≤2K<br>870K-1170K，≤3K<br>1170K-1370K，≤4K

Table S3. Summary of creep test conditions.

**Number**	**Conditions of tests**			
	**Temperature**	**Strain**	**Strain ratio**	**Hold time**
1	530K	0.8%	-1	60
2	530K	0.8%	-1	120
3	560K	0.6%	-1	60
4	560K	0.6%	-1	120
5	590K	0.5%	-1	60
6	590K	0.5%	-1	120
7	620K	0.4%	-1	60
8	620K	0.4%	-1	120
9	530K	0.8%	0	0
10	530K	0.8%	0	60
11	530K	0.8%	0	120
12	530K	0.5%	0	120
13	560K	0.6%	0	0
14	560K	0.6%	0	60
15	560K	0.6%	0	120
16	560K	0.4%	0	120
17	590K	0.5%	0	0
18	590K	0.5%	0	60
19	590K	0.5%	0	120
20	590K	0.3%	0	120
21	620K	0.4%	0	0
22	620K	0.4%	0	60

23	620K	0.4%	0	120	
24	620K	0.3%	0	120	

Table S4. Values of each parameter.

**Temperature(K)**	**300**	**300**	**500**	**500**	**530**	**530**
Rate of cycle (cpm)	20	20	20	20	20	20
Strain ratio	-1	-1	-1	-1	-1	-1
Strain range	1.6%	1.2%	1.6%	1.4%	0.8%	0.6%
$\Delta\varepsilon_p$	1.4E-3	1.3E-3	1.4E-3	1.5E-3	1.2E-3	1.0E-3
$\Delta\sigma$	236.84	224.08	230.56	214.42	192.46	185.26
m	3.54	1.79	1.42	1.38	2.80	2.07
k	2.07	3.54	2.95	3.66	2.07	2.71
a	-0.31	-0.30	-0.32	-0.25	-0.38	-0.35
c	-0.01	-0.02	-0.02	-0.01	-0.02	-0.001
Experiment value of ΔW_p	0.47	0.44	0.44	0.38	0.34	0.33
Calculation value of ΔW_p	0.44	0.40	0.42	0.35	0.35	0.34
Error	6.38%	9.09%	4.55%	7.89%	-2.94%	-3.03%
Experiment of N_f	4500	9547	3036	4008	3996	10052
Calculation of N_f	4764	8987	2839	3801	3732	9171
Error	-5.88%	5.86%	6.49%	5.17%	6.60%	8.76%
**Temperature(K)**	**560**	**560**	**590**	**590**	**620**	**620**
Rate of cycle (cpm)	20	20	20	20	20	20
Strain ratio	-1	-1	-1	-1	-1	-1
Strain range	0.8%	0.6%	0.6%	0.4%	0.4%	0.3%
$\Delta\varepsilon_p$	3.5E-3	2.8E-3	3.2E-3	2.5E-3	2.8E-3	2.4E-3
$\Delta\sigma$	181.24	176.42	152.24	147.68	120.46	114.52
m	1.30	2.78	1.95	2.49	2.29	1.05
k	2.43	3.58	3.12	3.11	2.82	3.07
a	-0.37	-0.31	-0.38	-0.34	-0.34	-0.21
c	-0.01	-0.02	-0.01	-0.02	-0.01	-0.01
Experiment value of ΔW_p	0.25	0.22	0.13	0.12	6.9E-2	5.9E-2
Calculation value of ΔW_p	0.25	0.24	0.14	0.12	7.0E-2	5.5E-2
Error	0%	-9.09%	-7.69%	0%	-1.45%	6.78%
Experiment of N_f	1644	4198	2327	6985	4055	13049
Calculation of N_f	1775	4454	2365	6617	3780	12912
Error	-7.97%	-6.09%	-1.62%	5.27%	6.77%	1.05%

Table S5. Specific test conditions and results.

**Number**	**Test condition**				**Creep fatigue life**
	**temperature**	**Strain range**	**Strain ratio**	**Holding time**	
**1**	530K	0.8%	-1	60	2954
2	530K	0.8%	-1	120	2437
3	560K	0.6%	-1	60	3849
4	560K	0.6%	-1	120	3561
5	590K	0.5%	-1	60	5734
6	590K	0.5%	-1	120	5293
7	620K	0.4%	-1	60	3816
8	620K	0.4%	-1	120	3578

9	530K	0.8%	0	0	3032
10	530K	0.8%	0	60	2785
11	530K	0.8%	0	120	2250
12	530K	0.5%	0	120	17657
13	560K	0.6%	0	0	3918
14	560K	0.6%	0	60	3657
15	560K	0.6%	0	120	3492
16	560K	0.4%	0	120	15728
17	590K	0.5%	0	0	5841
18	590K	0.5%	0	60	5548
19	590K	0.5%	0	120	5267
20	590K	0.3%	0	120	10574
21	620K	0.4%	0	0	3971
22	620K	0.4%	0	60	3782
23	620K	0.4%	0	120	3627
24	620K	0.3%	0	120	8956

Table S6. Comparison of training results in different groups.

Number	Training data		Test data	
	RMSE	R_a^2	RMSE	R_a^2
1	529.91	0.58	630.79	0.31
2	461.54	0.65	713.65	0.36
3	331.97	0.59	500.03	0.30
4	622.39	0.57	676.82	0.30
5	450.52	0.56	516.37	0.30
6	442.54	0.60	459.48	0.37
7	485.39	0.58	590.75	0.33

Table S7. Further treatment of evaluation indexes.

number	Training data		Test data		Mean value of RMSE	Mean value of R_a^2
	RMSE	R_a^2	RMSE	R_a^2		
**1**	**0.68**	0.17	0.67	0.24	0.68	0.21
2	0.45	1.00	1.00	0.89	0.73	0.95
3	0.00	0.30	0.16	0.00	0.08	0.15
4	1.00	0.01	0.86	0.02	0.93	0.02
5	0.41	0.00	0.22	0.10	0.32	0.05
6	0.38	0.45	0.00	1.00	0.19	0.73
7	0.53	0.21	0.52	0.44	0.53	0.33

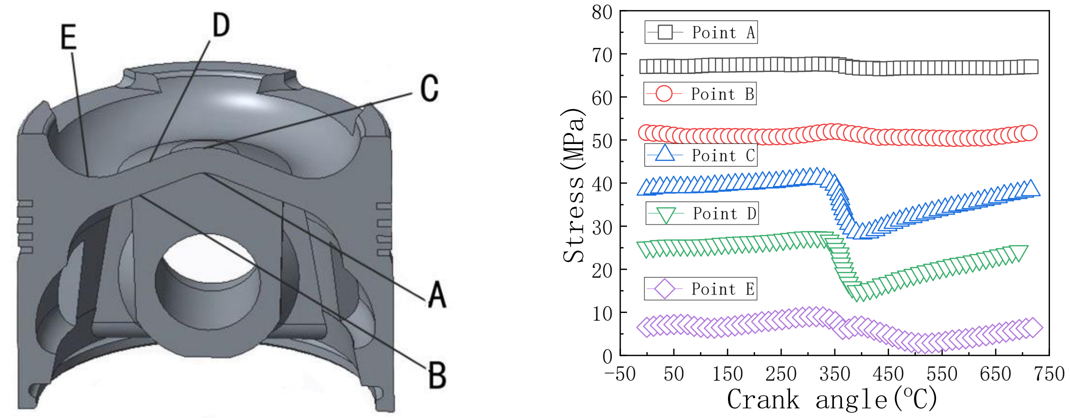

(a). Location diagram at different points

(b). Temperature fluctuation at different points

Figure S1. Change rule of thermal stresses at different positions with the crankshaft rotation angle

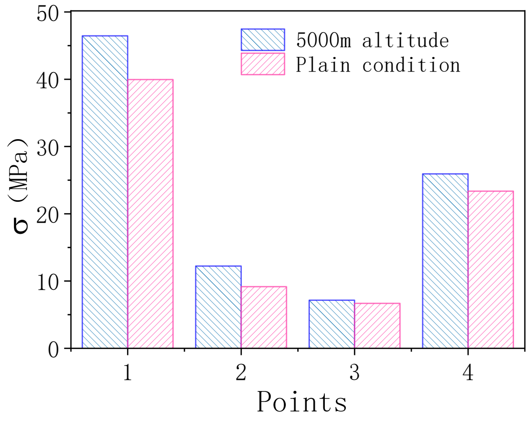

Figure S2. Piston equivalent stress amplitudes at different nodes

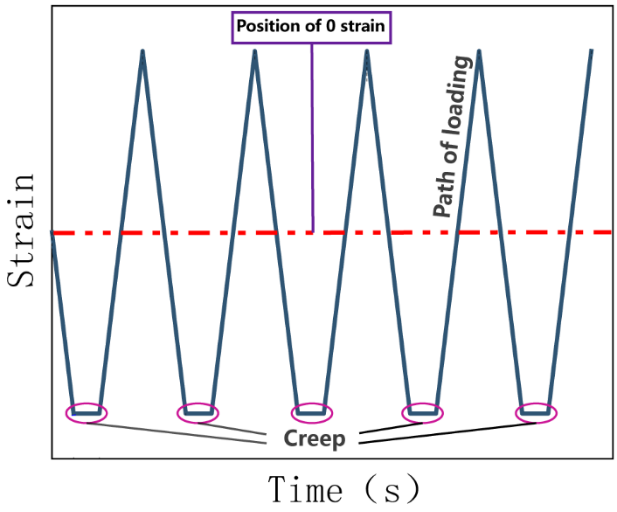

Figure S3. Loading waveform schematic diagram

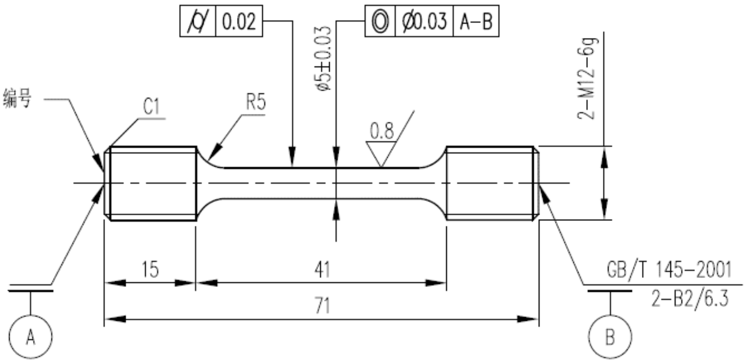

Figure S4. Processing requirements of specimens

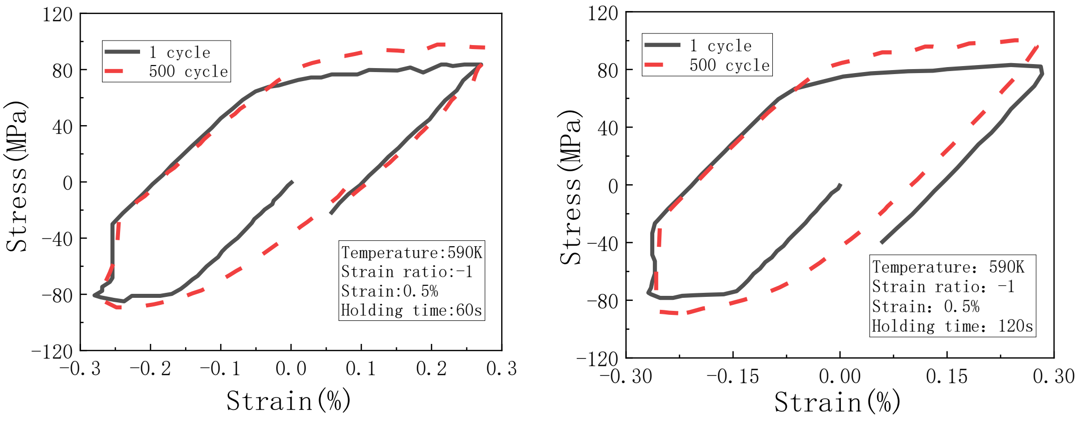

(a). 60s loading time at 590K

(b). 120s loading time at 590K

Figure S5. Results of different loading time at 590K

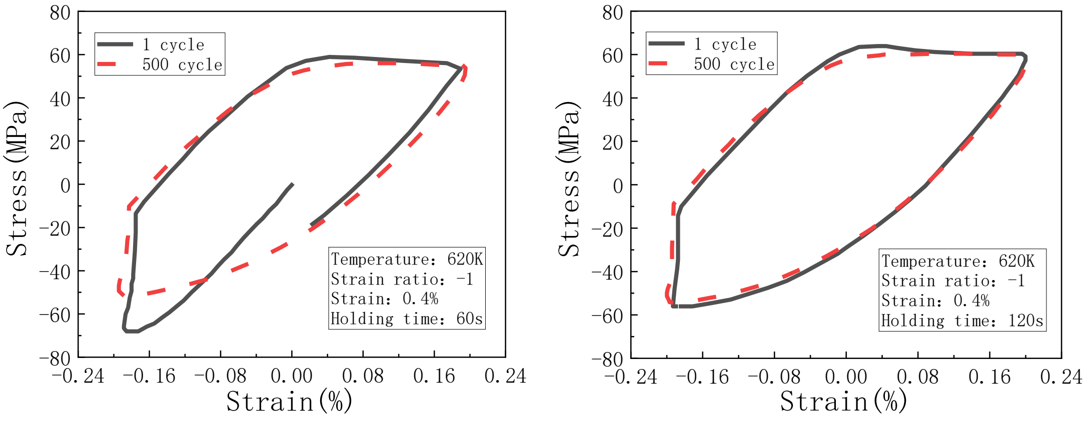

(a). 60s loading time at 620K

(b). 120s loading time at 620K

Figure S6. Results of different loading time at 620K
